# Genetic analysis of long-lived families reveals novel variants influencing high density-lipoprotein cholesterol

**DOI:** 10.3389/fgene.2014.00159

**Published:** 2014-06-03

**Authors:** Mary F. Feitosa, Mary K. Wojczynski, Robert Straka, Candace M. Kammerer, Joseph H. Lee, Aldi T. Kraja, Kaare Christensen, Anne B. Newman, Michael A. Province, Ingrid B. Borecki

**Affiliations:** ^1^Division of Statistical Genomics, Department of Genetics, Washington University School of MedicineSt. Louis, MO, USA; ^2^Department of Experimental and Clinical Pharmacology, College of Pharmacy, University of MinnesotaMinneapolis, MN, USA; ^3^Departments of Epidemiology and of Human Genetics, Center for Aging and Population Health University of PittsburghPittsburgh, PA, USA; ^4^Sergievsky Center and Taub Institute, College of Physicians and Surgeons, Columbia UniversityNew York, NY, USA; ^5^The Danish Aging Research Center, Epidemiology, University of Southern DenmarkOdense, Denmark; ^6^Departments of Clinical Genetics and Clinical Biochemistry and Pharmacology, Odense University HospitalOdense, Denmark; ^7^Department of Epidemiology, University of Pittsburgh Graduate School of Public HealthPittsburgh, PA, USA

**Keywords:** *NALP1*, lipids, genomewide association study, aging, familial longevity, family-based study

## Abstract

The plasma levels of high-density lipoprotein cholesterol (HDL) have an inverse relationship to the risks of atherosclerosis and cardiovascular disease (CVD), and have also been associated with longevity. We sought to identify novel loci for HDL that could potentially provide new insights into biological regulation of HDL metabolism in healthy-longevous subjects. We performed a genome-wide association (GWA) scan on HDL using a mixed model approach to account for family structure using kinship coefficients. A total of 4114 subjects of European descent (480 families) were genotyped at ~2.3 million SNPs and ~38 million SNPs were imputed using the 1000 Genome Cosmopolitan reference panel in MACH. We identified novel variants near-*NLRP1* (17p13) associated with an increase of HDL levels at genome-wide significant level (*p* < 5.0E-08). Additionally, several *CETP* (16q21) and *ZNF259-APOA5-A4-C3-A1* (11q23.3) variants associated with HDL were found, replicating those previously reported in the literature. A possible regulatory variant upstream of *NLRP1* that is associated with HDL in these elderly Long Life Family Study (LLFS) subjects may also contribute to their longevity and health. Our *NLRP1* intergenic SNPs show a potential regulatory function in Encyclopedia of DNA Elements (ENCODE); however, it is not clear whether they regulate *NLRP1* or other more remote gene. *NLRP1* plays an important role in the induction of apoptosis, and its inflammasome is critical for mediating innate immune responses. Nlrp1a (a mouse ortholog of human *NLRP1*) interacts with SREBP-1a (17p11) which has a fundamental role in lipid concentration and composition, and is involved in innate immune response in macrophages. The *NLRP1* region is conserved in mammals, but also has evolved adaptively showing signals of positive selection in European populations that might confer an advantage. *NLRP1* intergenic SNPs have also been associated with immunity/inflammasome disorders which highlights the biological importance of this chromosomal region.

## Introduction

Epidemiologic studies have shown that high plasma levels of high-density lipoprotein cholesterol (HDL) have protective effects on atherosclerosis and cardiovascular disease (CVD) across multiple populations (Toth et al., 2013; Feig et al., 2014). HDL seems to contribute to atheroprotection as an anti-inflammatory, and is involved in a myriad of biologic processes, such as: promoting reverse cholesterol transport, regulating plasma membrane cholesterol content, mediating nitric oxide production in the endothelium, inhibiting low-density lipoprotein cholesterol oxidation, inhibiting the expression of proinflammatory cell adhesion molecules on endothelial cell apoptosis, and modulating the activity of macrophage chemotactic factors (Toth et al., 2013; Feig et al., 2014). Recently, Feig et al. (2014) demonstrated evidence, from preclinical and clinical studies, that HDL can promote the regression of atherosclerosis when the levels of functional HDL particles are increased, either by stimulating endogenous production of (lipid-poor) apoAI or by providing HDL or apoAI exogenously. In addition, there is evidence that HDL levels are associated with longevity (Bergman et al., 2007; Koropatnick et al., 2008; Newman et al., 2011; Rahilly-Tierney et al., 2011). A previous study from the Long Life Family Study (LLFS), which selected families characterized as healthy and having a strong history of longevity, showed that probands and offspring had higher HDL levels and lower cardiovascular risk factors as compared to similar aged individuals in the Cardiovascular Health Study (Newman et al., 2011). Prospective studies have also found that HDL levels in subjects who had survived to exceptional age were higher than those of their younger counterparts (Koropatnick et al., 2008; Rahilly-Tierney et al., 2011). These studies suggest that high levels of HDL may contribute to exceptional longevity, likely due in part to reduction in CVD risk.

Genome-wide association (GWA) studies have allowed the identification of many genetic loci that influence plasma levels of HDL (e.g., Teslovich et al., 2010; Brautbar et al., 2011); however, the proportion of variance for HDL explained by these loci remains low. We sought to identify genetic variants influencing HDL levels in a unique sample of families of exceptionally healthy, elderly people from the LLFS, and to attempt to annotate the function of any associated variants using the Encyclopedia of DNA Elements (ENCODE) resources.

## Materials and methods

### Study design

The LLFS (https://dsgweb.wustl.edu/llfs/) was designed to determine genetic, behavioral, and environmental factors related to families of exceptionally healthy, elderly individuals. Families were sampled from four clinical centers: Boston University Medical Center in Boston MA, Columbia College of Physicians and Surgeons in New York City NY, the University of Pittsburgh in Pittsburgh PA, USA, and University of Southern Denmark, Denmark. The study characteristics, recruitment, eligibility and enrollment have been previously described (Pedersen et al., 2006; Sebastiani et al., 2009; Newman et al., 2011). In brief, the LLFS recruited selected families with multiple exceptionally old living individuals, totaling 4559 individuals, which included long-lived probands and their siblings (*n* = 1445), their offspring (*n* = 2329) and spouse controls (*n* = 785). The probands were at least 79 years old in the USA centers, and 90 years old or above in Denmark. The families were selected to participate in the study based on The Family Longevity Selection Score (FLoSS) (Sebastiani et al., 2009) which calculated the rank sibships by current age or age at death of siblings, the size of the sibship and the number of alive individuals available for study. A proband's family was FLoSS eligible if reached a score of 7 or higher, which met the following criteria: (1) the proband, at least one living sibling, and one of their living offspring (minimum family size of 3) were all able to give informed consent, and (2) were willing to participate in the interview and examination including the blood sample for serum and DNA extraction. A broad range of phenotypes were assessed, such as: validated age (by driver's license, birth certificate, or other official document or source), anthropometric measures, blood pressure, lipids, glucose metabolism, lung function, physical, and performance functions (e.g., difficulty with activities of daily living, instrumental activities of daily living, mobility, gait speed, chair stands and standing balance, and strength), cognitive testing, education, behavior (e.g., smoking and alcohol intake), and history of disease (e.g., heart disease, stroke, hypertension, diabetes, chronic lung disease, peripheral artery disease, and cancer) among other traits. Reported medications, including anti-hypertensives, anti-anginals, oral hypoglycemics and insulin, and lipid lowering drugs, were confirmed by medication inventory of all prescriptions and over-the-counter medications taken in the past 2 weeks.

### Phenotype

HDL was measured directly in serum using the Roche HDL 3rd generation direct method (Roche Diagnostics, Indianapolis, IN 46250) on a Roche Modular P Chemistry Analyzer (Roche Diagnostics Corporation).

### Genotype

The genotype data included ~2.3 million SNPs from the Illumina Omni chip. Quality control was performed before imputation by checking pedigree relationships using GRR (Abecasis et al., 2001) and Loki (Heath, 1997) approaches. Single-nucleotide polymorphism (SNPs) were eliminated if presented Mendelian errors, coded allele frequency <1% or >99%, deviations from Hardy-Weinberg equilibrium (*p* < 1.0 × 10^−6^), and/or low call rate (98%). Approximately 2 million autosomal SNPs remained after genotype quality control. Imputation was performed on phased 1000 Genomes with Cosmopolitan data as a reference (version 2010-11 data freeze, 2012-03-04 haplotypes; http://www.sph.umich.edu/csg/abecasis/MaCH/download/1000G.2012-03-14.html) and using MACH and MINIMAC to perform the imputation (http://www.sph.umich.edu/csg/abecasis/mach/). This process led to a hybrid dataset with 38,245,546 SNPs, of which 2,225,338 SNPs were genotyped and 36,020,208 SNPs were imputed.

### Covariate adjustment

Cryptic stratification was accounted for by estimating the first 20 principal components (PCs, EIGENSTRAT (Price et al., 2006) in genotype data with the highest call rates in 1522 independent individuals. The PC models were then applied to the remaining (non-independent) family members. HDL levels were adjusted using two steps: (1) forcing field centers into the regression analysis, and then (2) adding age, age^2^, and twenty PCs within sex groups using a stepwise regression analysis and retaining terms significant at the 5% level. Residuals were normalized to have mean 0 and standard deviation 1, and normalized residuals were used as phenotypes to test for genotype-phenotype association.

### Association analysis

GWA scan was carried out assuming additive effects with mixed model linear regression, accounting for dependency among family members as a function of their kinship correlations (R kinship package: http://cran.r-project.org/web/packages/GWAF/). Results were filtered to include SNPs with acceptable imputation quality (r^2^ MACH > 0.3) and with effect allele frequency between 1–99%. After genotype data filtering, a total of ~9.4 M SNPs were taken in the GWA results.

### Regional plot

Regional plots were generated with LocusZoom (Pruim et al., 2010) for investigation of linkage disequilibrium (LD) and block structure based on hg19/1000 Genomes March 2012-EUR.

### Bionformatic analyses

To investigate the functions of our candidate SNPs (Table 1), such as, type-specific patterns of promoters and enhancers, and their regulatory motif enrichment and regulator expression, we used the ENCODE Consortium (https://genome.ucsc.edu/ENCODE/), the Roadmap Epigenome Mapping Consortium (http://www.ppmroadmap.com/) and the Single Nucleotide Polymorphism Database (dbSNP, https://www.ncbi.nlm.nih.gov/SNP/), as implemented in HaploReg (v2, www.broadinstitute.org/mammals/haploreg/; Ernst et al., 2011) and RegulomeDB (www.regulomedb.org/; Boyle et al., 2012).

**Table 1:** **Characteristics of the variables studied in the GWA analysis**.

**Variables**	**Men**	**Women**
Sample size (%)	1858 (45.2)	2256
Age (years)	70.8 ± 15.2	69.6 ± 16.0
Body mass index (kg/m^2^)	27.5 ± 4.1	26.7 ± 5.3
Fasting levels of HDL (mg/dL)	52.6 ± 15.1	64.5 ± 17.2
Lipid-lowering medication (%)	40.4	35.0

*Variables are shown as number (N), and/or frequency (%), or mean ± standard deviation*.

## Results

Table 1 shows the characteristics of 4114 subjects of European-American individuals (480 families) from the LLFS that participated in the GWA analyses. The mean levels of age and BMI were not significantly different in men as compared to women. However, the proportion of subjects taking lipid-lowering medications in men is higher than in women, while the mean levels of fasting plasma levels of HDL are higher in women than men. The distribution of mean levels of HDL in the sex-combined data is depicted in Supplementary Figure 1.

The estimated GWA inflation (λ_GC_) for HDL was 1.03, indicating that there was no significant population stratification. Also, there were results that exceeded the genome-wide significant threshold (*p* < 5.0E-08), suggesting the effect of variants influencing HDL levels (Supplementary Figure 2).

**Figure 1:**
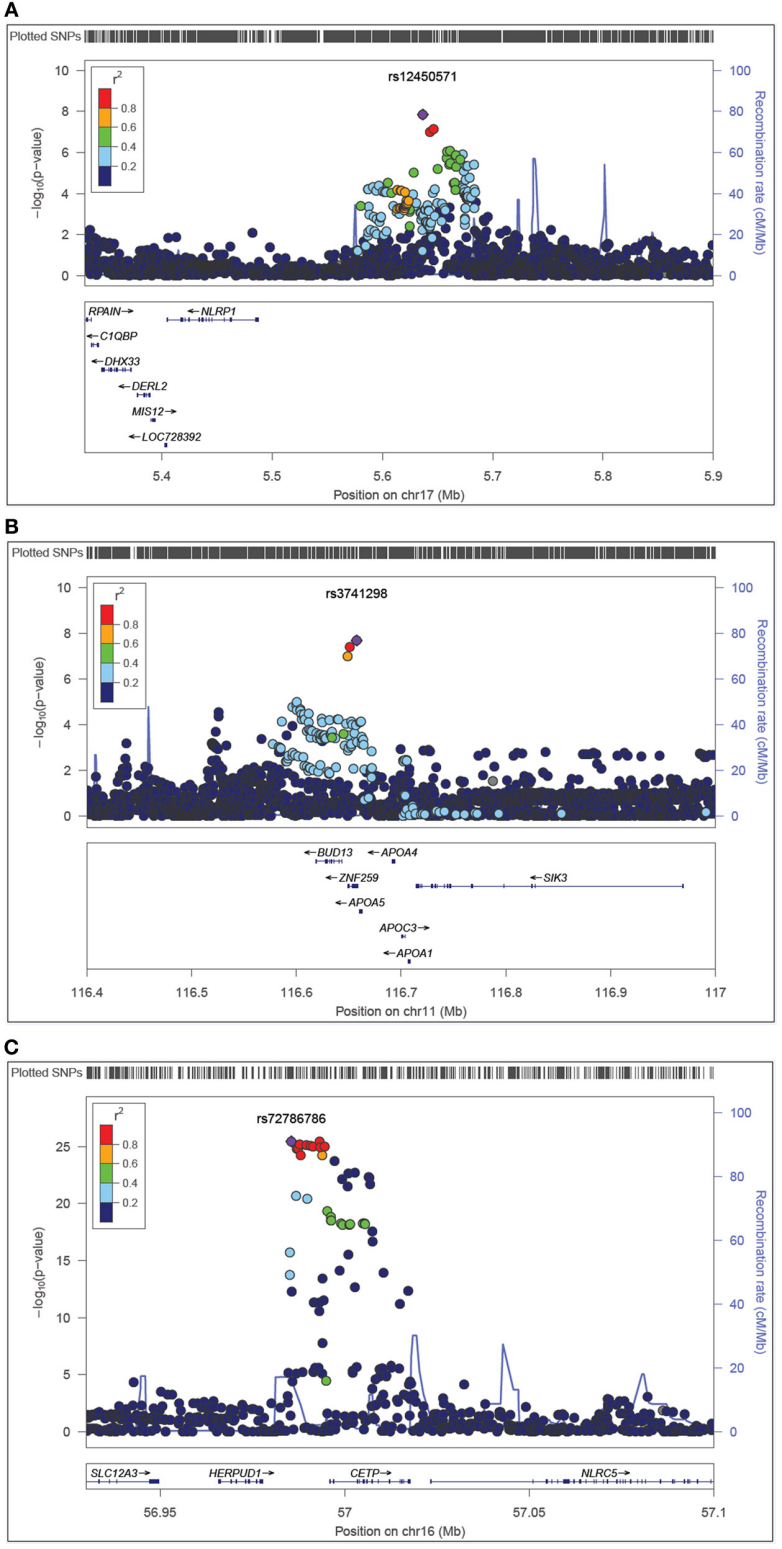
**Regional plots represent the associations between plasma levels of HDL and *NLRP1* (A), *ZNF259* (B), and *CEPT* (C) gene regions**. The −log_10_ (*p*-value) for HDL is represented in the ordinate axis, while the chromosome position in Megabase (Mb) using build 37.3 is represented in the abscissa axis. The bottom panels give the relative positions of genes in the locus. Purple diamond indicates the effect of the top significant SNP with HDL cholesterol. Each circle indicates a SNP with the color of the circle representing the correlation (*r*^2^) between that SNP and the top significant SNP. Blue line indicates estimated recombination hotspots.

On chromosome 17p13, we found statistical evidence for a novel variant near-*NLRP1* (rs12450571: *p* = 1.82E-08, β = 0.13, minor allele frequency (maf) of G allele=0.47) influencing fasting plasma levels of HDL. In addition, we detected association of variants in *ZNF259-APOA5-A4-C3-A1* [11q23.3, rs3741298: *p* = 2.04E-08, β = −0.16, maf(C) = 0.21] and *HERPUD1*-*CEPT* (16q21, rs72786786: *p* = 3.64E-26, β = −0.28, maf (A) = 0.32] with HDL, which were previously reported in the literature (e.g., Teslovich et al., 2010; Brautbar et al., 2011). The regional plots show the LD and block structures for SNPs on 17p13 (Figure 1A), on 11q23.3 (Figure 1B) and on 16q21 (Figure 1C). Table 2 shows the SNPs with the strongest associations with HDL levels in each chromosome region, while the Supplementary Table 1 lists all associated SNPs.

**Table 2:** **GWA results for the most significant SNP (*p* < 5.0E-08) at each locus for plasma levels of HDL**.

**SNP**	**Chr**	**Location**	**CA**	**CAF**	**IP**	**Gene**	**Function**	***N***	**β**	***SE***	***P***
rs3741298	11	116657561	T	0.79	1	ZNF259	intron	4114	0.16	0.03	2.04E-08
rs12450571	17	5636654	G	0.47	0	near-NLRP1	4110		0.13	0.02	1.82E-08
rs72786786	16	56985514	A	0.32	1	near-CETP	4114		0.28	0.03	3.64E-26

*Chr, chromosome; Location, in Megabase using build 37.3; CA, coded allele; CAF, coded allele frequency; IP, Imputed SNP is represented as “Y” (“N” = typed); β, regression coefficient for the coded allele; SE, Standard error of the β P, P value of association*.

### Bionformatics

Using the ENCODE, Roadmap and dbSNP data in RegulomeDB and HaploReg, we assessed the annotations of the novel findings near-*NLRP1* on 17p13 (Supplementary Table 2). The variants rs12450571, rs8080616, and rs2215496 were suggested to have a role in binding motif (SEF-1, Foxj1, Foxa, Foxk1, Foxo, Foxp1, HDZC2, HMG, Irf, Nanog, Sox, p300, and/or Pou2f2; Matys et al., 2006; Badis et al., 2009; Scharer et al., 2009), and in methylating histones (H3k09me3, H4k20me1, H3k27me3, H2az, and H3k9me1). The rs12450571 SNP was also suggested to interact with chromatin remodeling in cell lines (HUES6, ES-I3, and iPS-15b). The *ZNF259-*rs3741298 and *HERPUD1*-*CEPT*-rs72786786 were implicated in ENCODE DNAse and regulatory chromatin states associated with diseases, in binding proteins, in histone methylations and in motif changes (Supplementary Table 2). These annotations suggest that the HDL-associated variants found in the LLFS subjects are involved in genetic regulatory functions.

## Discussion

Our current study, focusing on GWA, demonstrated evidence of genes associated with fasting plasma levels of HDL cholesterol, which have been linked to inflammation, apoptosis and/or longevity (Barzilai et al., 2006; Bergman et al., 2007; Jin et al., 2007; Magitta et al., 2009; Sanders et al., 2010; Zurawek et al., 2010; Dieudé et al., 2011). We have identified novel variants near-*NLRP1* (NLR family, pyrin domain containing 1; 17p13) associated with an increase in HDL levels, and also replicated associations for HDL with several established variants in *HERPUD1*-*CEPT* (16q21) and *ZNF259-APOA5-A4-C3-A1* (11q23.3) (Teslovich et al., 2010; Brautbar et al., 2011). The variants in *CEPT* (cholesteryl ester transfer protein) and *APOC3* (apolipoprotein C3) genes have been connected with healthy aging, in addition to associations with HDL levels. *CETP* V405 homozygosity was associated with slower memory decline and lower incidence of dementia and Alzheimer disease risk in healthy older adults compared with controls in the Einstein Aging Study (Sanders et al., 2010). In Ashkenazi Jews from the Longevity Gene Study, high levels of HDL and its large lipoprotein sizes were over represented in centenarians, as well as the prevalence of homozygosity for I405V-*CETP* and 641C-*APOC3* in both centenarians and their offspring than in the controls (Barzilai et al., 2006; Bergman et al., 2007). We also found high levels of HDL and a borderline higher prevalence of homozygosity for 641C-*APOC3* (rs2542052: *p* = 0.06) in the healthy LLFS subjects as compared to an independent data from the Family Heart Study (*N* = 3794 European-Americans) that has approximately half families CVD-selected and the other half families randomly-selected. While the findings have reproducibly demonstrated that HDL levels are high in healthy-longevous populations, we have only begun to identify the underlying genetic factors.

Association of multiple variants in the intergenic region and within *NLRP1* have been reported to be associated with innate immunity/inflammasome disorders, including vitiligo (Jin et al., 2007), Addison's disease (Zurawek et al., 2010), systemic sclerosis -related pulmonary fibrosis (Dieudé et al., 2011), and type 1 diabetes (Magitta et al., 2009). It is interesting to note that some of the *NLRP1*-intergenic SNPs for vitiligo and other diseases [including systemic lupus erythematosus (SLE); (Jin et al., 2007)] also showed suggestive associations for HDL in LLFS (e.g., rs995298: *p* = 7.09E-06, rs8065677: *p* = 2.98E-04, rs2716900: *p* = 4.28E-04). These SNPs are in moderate LD (*r*^2^ = 0.30 – 0.57) with the HDL-associated SNPs (rs12450571, rs8080616, and rs2215496) reported in the present study, suggesting that this expanded region may be relevant to these multiple phenotypes. It is widely recognized that low HDL levels are risk factors for atherosclerosis, which is also a major co-morbid condition in autoimmune diseases (Skaggs et al., 2010). Low HDL levels and insulin resistance were significantly increased in patients with vitiligo (Karadag et al., 2011), and a dysfunctional, pro-inflammatory form of HDL (piHDL) was present in 45% of SLE patients vs. 4% of controls (McMahon et al., 2006).

There is also evidence linking *NLRP1* to longevity (Flachsbart et al., 2010), Alzheimer disease (Pontillo et al., 2012), and CVD (Garg, 2011), suggesting it interacts with other lipid related-genes (Sanz et al., 2004; Im et al., 2011). A study demonstrated that SREBP-1a (sterol regulatory element binding proteins, 17p11) links lipid metabolism to the macrophage innate immune response through Nlrp1a, a mouse ortholog of human *NLRP1* (Im et al., 2011). SREBP-1a is required for lipopolysaccharide-stimulated interleukin 1β (IL1β) production, which occurs by SREBP-1a activating the expression of the Nlrp1a gene through a binding site in its proximal promoter. SREBP-1a proteins belong to a small family of transcription factors and are key regulators of cellular lipid levels. Im et al. (2011) proposed that SREBP-1a evolved to directly regulate genes of the innate immune response in macrophages because cell proliferation and membrane expansion/ rearrangements are both essential in the macrophage response to pathogen challenge and both require new lipid synthesis. The lipid levels are critical for cell-environment interactions in macrophages, and regulation of lipid concentration and composition is fundamental to optimize protein-lipid microenvironments required for transport, signaling, internalization, and shape alterations such as blebbing and invagination. Other studies in myeloid leukaemia cells proposed that *NLRP1* and CREB (cAMP-response-element-binding protein) may contribute by modulating the response to pro-inflammatory stimuli. *NLRP1* was found to be transcriptionally regulated by CREB transcription factor in myeloid cells (Sanz et al., 2004). CREB has also been associated with COX-2 (cyclooxygenase-2) expression, which in turn is induced by HDL in vascular endothelial cells through a bioactive lysophospholipid SphK-2 (sphingosine kinase-2) (Xiong et al., 2014). The COX-2 enzyme is responsible for inflammation and pain and has been reported to exert cardioprotective effects in a model of myocardial ischemia–reperfusion injury via activating PGI-2 (prostaglandin I-2) synthesis (Bolli et al., 2002). These findings may suggest that all these components, including *NLRP1* gene region, possibly contribute to HDL anti-atherogenic effects; however, the mechanisms involved remain largely unknown and more in-depth investigations are needed.

The *NLRP1* (also called as *NALP1*) is a member of NLR (nucleotide-binding domain leucine-rich repeat containing) multi-domain proteins family of intracellular sensors. *NLRP1* encodes a protein that contains a N-terminal pyrin-like motif, which may be involved in protein-protein interactions and play a role in regulating the apoptotic machinery. *NLRP1* recruits the adapter protein ASC (apoptosis-associated speck-like protein containing CARD), caspase 1, and caspase 5 to a complex termed the *NLRP1* inflammasome, which activates the proinflammatory cytokine, pro-IL1β. Pyrin-like motif is conserved with other mammalian proteins such as ASC, and is evolutionarily conserved across other species, as seen in sequence alignments with zebrafish ASC1 (Hlaing et al., 2001). In various human populations, Vasseur et al. (2012) studied germline-encoded microbial sensors characterizing the levels of genetic sequence diversity of the central nucleotide-binding domain termed NACHT. The authors identified *NLRP1* amino acid changes that conferred a positive-selective advantage related to microbial sensing in Europe, Africa, and Asia. Also, they detected another *NLRP1* positive-selection event restricted to Europe. Some of the selective advantage *NLRP1* variants have shown association with various autoimmune diseases, including Addison's disease, type I diabetes, and vitiligo, which may suggest that variants in the intergenic region and within *NLRP1* may cause differences in susceptibility to infections (Hlaing et al., 2001; Vasseur et al., 2012) and immunity-related disorders (Jin et al., 2007).

ENCODE annotation of our novel near-*NLRP1* variants (rs12450571, rs8080616, and rs2215496) associated with HDL levels suggested they may have a regulatory function (Supplementary Table 2). Glinskii et al. (2009) studied intergenic trans-regulatory RNAs containing a disease-linked SNP sequence and demonstrated that another *NLRP1* intergenic SNP (rs2670660) was expressed in human cells, encoding a small RNA. The expression of rs2670660-encoded transRNAs triggered concomitant activation of the polycomb pathway genes which catalyzed histone H3K27me3. Rs2670660 alters predicted binding motifs for the transcription factors HMGA1 [HMG-I(Y)] and MYB. Although our near-*NLRP1* SNPs are in very low LD (*r*^2^ < 0.04) with rs2670660, there are other reported associated SNPs in the expanded *NLRP1* region that may have regulatory function. It is worthy to mention that our near-*NLRP1* SNPs were also suggested to participate in histone methylation (H3k09me3, H4k20me1, H3k27me3, H2az, and H3k9me1) and as a binding motif (rs8080616) for HMG (high-mobility group). While further studies are needed, these data suggest that this is a conserved regulatory region that may influence *NLRP1* or other genes residing near 17p13.

Our study has several relevant strengths and limitations. It is a large family study of exceptionally healthy and well-characterized longevous subjects. Data published from the Global Lipid Genetic Consortium did not report SNPs meeting genomewide criterion influencing HDL in this region, thus, our novel finding of *NLRP1* intergenic SNPs associated with HDL requires replication. Despite the resources for annotation available, it is not clear whether our HDL-associated intergenic SNPs regulate *NLRP1* or even other more remote genes. Our SNPs, however, are in moderate LD with *NLRP1* intergenic SNPs that were associated with immunity/inflammasome disorders, which underscores the biological importance of this region. There is also published evidence that *NLRP1* interacts with genes (SREBP-1a and CREB) likely associated with lipid metabolism, apoptosis, autoimmune and/or autoinflammatory diseases, factors that likely influence HDL levels and longevity. Thus, our findings may provide new insights into the biological regulation of HDL metabolism and longevity that deserve further investigations.

### Conflict of interest statement

The authors declare that the research was conducted in the absence of any commercial or financial relationships that could be construed as a potential conflict of interest.

## References

[B1] Abecasis G. R. Cherny S. S. Cookson W. O. Cardon L. R. (2001). GRR: graphical representation of relationship errors. Bioinformatics 17, 742–743. 10.1093/bioinformatics/17.8.742 11524377

[B2] Badis G. Berger M. F. Philippakis A. A. Talukder S. Gehrke A. R. Jaeger S. A.. (2009). Diversity and complexity in DNA recognition by transcription factors. Science 324, 1720–1723. 10.1126/science.1162327 19443739 PMC2905877

[B3] Barzilai N. Atzmon G. Derby C. A. Bauman J. M. Lipton R. B. (2006). A genotype of exceptional longevity is associated with preservation of cognitive function. Neurology 67, 2170–2175. 10.1212/01.wnl.0000249116.50854.65 17190939

[B4] Bergman A. Atzmon G. Ye K. MacCarthy T. Barzilai N. (2007). Buffering mechanisms in aging: a systems approach toward uncovering the genetic component of aging. PLoS Comput. Biol. 3:e170. 10.1371/journal.pcbi.0030170 17784782 PMC1963511

[B5] Bolli R. Shinmura K. Tang X. L. Kodani E. Xuan Y. T. Guo Y.. (2002). Discovery of a new function of cyclooxygenase (COX)-2: COX-2 is a cardioprotective protein that alleviates ischemia/reperfusion injury and mediates the late phase of preconditioning. Cardiovasc. Res. 55, 506–519. 10.1016/S0008-6363(02)00414-5 12160947 PMC3242376

[B6] Boyle A. P. Hong E. L. Hariharan M. Cheng Y. Schaub M. A. Kasowski M.. (2012). Annotation of functional variation in personal genomes using RegulomeDB. Genome Res. 22, 1790–1797. 10.1101/gr.137323.112 22955989 PMC3431494

[B7] Brautbar A. Covarrubias D. Belmont J. Lara-Garduno F. Virani S. S. Jones P. H.. (2011). Variants in the APOA5 gene region and the response to combination therapy with statins and fenofibric acid in a randomized clinical trial of individuals with mixed dyslipidemia. Atherosclerosis 219, 737–742. 10.1016/j.atherosclerosis.2011.08.015 21889769 PMC6174528

[B8] Dieudé P. Guedj M. Wipff J. Ruiz B. Riemekasten G. Airo P.. (2011). NLRP1 influences the systemic sclerosis phenotype: a new clue for the contribution of innate immunity in systemic sclerosis-related fibrosing alveolitis pathogenesis. Ann. Rheum. Dis. 70, 668–674. 10.1136/ard.2010.131243 21149496

[B9] Ernst J. Kheradpour P. Mikkelsen T. S. Shoresh N. Ward L. D. Epstein C. B.. (2011). Mapping and analysis of chromatin state dynamics in nine human cell types. Nature 473, 43–49. 10.1038/nature09906 21441907 PMC3088773

[B10] Feig J. E. Hewing B. Smith J. D. Hazen S. L. Fisher E. A. (2014). High-density lipoprotein and atherosclerosis regression: evidence from preclinical and clinical studies. Circ. Res. 114, 205–213. 10.1161/CIRCRESAHA.114.300760 24385513 PMC3918097

[B11] Flachsbart F. Franke A. Kleindorp R. Caliebe A. Blanche H. Schreiber S.. (2010). Investigation of genetic susceptibility factors for human longevity - a targeted nonsynonymous SNP study. Mutat. Res. 694, 13–19. 10.1016/j.mrfmmm.2010.08.006 20800603

[B12] Garg N. J. (2011). Inflammasomes in cardiovascular diseases. Am. J. Cardiovasc. Dis. 1, 244–254. 22254202 PMC3253520

[B13] Glinskii A. B. Ma J. Ma S. Grant D. Lim C. U. Sell S.. (2009). Identification of intergenic trans-regulatory RNAs containing a disease-linked SNP sequence and targeting cell cycle progression/differentiation pathways in multiple common human disorders. Cell Cycle 8, 3925–3942. 10.4161/cc.8.23.10113 19923886

[B14] Heath S. C. (1997). Markov chain Monte Carlo segregation and linkage analysis for oligogenic models. Am. J. Hum. Genet. 61, 748–760. 10.1086/515506 9326339 PMC1715966

[B15] Hlaing T. Guo R. F. Dilley K. A. Loussia J. M. Morrish T. A. Shi M. M.. (2001). Molecular cloning and characterization of DEFCAP-L and -S, two isoforms of a novel member of the mammalian Ced-4 family of apoptosis proteins. J. Biol. Chem. 276, 9230–9238. 10.1074/jbc.M009853200 11076957

[B16] Im S. S. Yousef L. Blaschitz C. Liu J. Z. Edwards R. A. Young S. G.. (2011). Linking lipid metabolism to the innate immune response in macrophages through sterol regulatory element binding protein-1a. Cell Metab. 13, 540–549. 10.1016/j.cmet.2011.04.001 21531336 PMC3090630

[B17] Jin Y. Mailloux C. M. Gowan K. Riccardi S. L. LaBerge G. Bennett D. C.. (2007). NALP1 in vitiligo-associated multiple autoimmune disease. N. Engl. J. Med. 356, 1216–1225. 10.1056/NEJMoa061592 17377159

[B18] Karadag A. S. Tutal E. Ertugrul D. T. (2011). Insulin resistance is increased in patients with vitiligo. Acta Derm. Venereol. 91, 541–544. 10.2340/00015555-1141 21597678

[B19] Koropatnick T. A. Kimbell J. Chen R. Grove J. S. Donlon T. A. Masaki K. H.. (2008). A prospective study of high-density lipoprotein cholesterol, cholesteryl ester transfer protein gene variants, and healthy aging in very old Japanese-american men. J. Gerontol. A Biol. Sci. Med. Sci. 63, 1235–1240. 10.1093/gerona/63.11.1235 19038839

[B20] Magitta N. F. Bøe Wolff A. S. Johansson S. Skinningsrud B. Lie B. A. Myhr K. M.. (2009). A coding polymorphism in NALP1 confers risk for autoimmune Addison's disease and type 1 diabetes. Genes Immun. 10, 120–124. 10.1038/gene.2008.85 18946481

[B21] Matys V. Kel-Margoulis O. V. Fricke E. Liebich I. Land S. Barre-Dirrie A.. (2006). TRANSFAC and its module TRANSCompel: transcriptional gene regulation in eukaryotes. Nucleic Acids Res. 34, D108–D110. 10.1093/nar/gkj143 16381825 PMC1347505

[B22] McMahon M. Grossman J. FitzGerald J. Dahlin-Lee E. Wallace D. J. Thong B. Y.. (2006). Proinflammatory high-density lipoprotein as a biomarker for atherosclerosis in patients with systemic lupus erythematosus and rheumatoid arthritis. Arthritis. Rheum. 54, 2541–2549. 10.1002/art.21976 16868975

[B23] Newman A. B. Glynn N. W. Taylor C. A. Sebastiani P. Perls T. T. Mayeux R.. (2011). Health and function of participants in the long life family study: a comparison with other cohorts. Aging (Albany, NY) 3, 63–76. 21258136 10.18632/aging.100242 PMC3047140

[B24] Pedersen C. B. Gotzsche H. Moller J. O. Mortensen P. B. (2006). The danish civil registration system. A cohort of eight million persons. Dan. Med. Bull. 53, 441–449. 17150149

[B25] Pontillo A. Catamo E. Arosio B. Mari D. Crovella S. (2012). NALP1/NLRP1 genetic variants are associated with Alzheimer disease. Alzheimer Dis. Assoc. Disord. 26, 277–281. 10.1097/WAD.0b013e318231a8ac 21946017

[B26] Price A. L. Patterson N. J. Plenge R. M. Weinblatt M. E. Shadick N. A. Reich D. (2006). Principal components analysis corrects for stratification in genome-wide association studies. Nat. Genet. 38, 904–909. 10.1038/ng1847 16862161

[B27] Pruim R. J. Welch R. P. Sanna S. Teslovich T. M. Chines P. S. Gliedt T. P.. (2010). LocusZoom: regional visualization of genome-wide association scan results. Bioinformatics 26, 2336–2337. 10.1093/bioinformatics/btq419 20634204 PMC2935401

[B28] Rahilly-Tierney C. R. Spiro A. 3rd Vokonas P. Gaziano J. M. (2011). Relation between high-density lipoprotein cholesterol and survival to age 85 years in men (from the VA normative aging study). Am. J. Cardiol. 107, 1173–1177. 10.1016/j.amjcard.2010.12.015 21296318

[B29] Sanders A. E. Wang C. Katz M. Derby C. A. Barzilai N. Ozelius L.. (2010). Association of a functional polymorphism in the cholesteryl ester transfer protein (CETP) gene with memory decline and incidence of dementia. JAMA 303, 150–158. 10.1001/jama.2009.1988 20068209 PMC3047443

[B30] Sanz C. Calasanz M. J. Andreu E. Richard C. Prosper F. Fernandez-Luna J. L. (2004). NALP1 is a transcriptional target for cAMP-response-element-binding protein (CREB) in myeloid leukaemia cells. Biochem J. 384(Pt 2), 281–286. 10.1042/BJ20040867 15285719 PMC1134111

[B31] Scharer C. D. McCabe C. D. Ali-Seyed M. Berger M. F. Bulyk M. L. Moreno C. S. (2009). Genome-wide promoter analysis of the SOX4 transcriptional network in prostate cancer cells. Cancer Res. 69, 709–717. 10.1158/0008-5472.CAN-08-3415 19147588 PMC2629396

[B32] Sebastiani P. Hadley E. C. Province M. Christensen K. Rossi W. Perls T. T.. (2009). A family longevity selection score: ranking sibships by their longevity, size, and availability for study. Am. J. Epidemiol. 170, 1555–1562. 10.1093/aje/kwp309 19910380 PMC2800272

[B33] Skaggs B. J. Hahn B. H. Sahakian L. Grossman J. McMahon M. (2010). Dysfunctional, pro-inflammatory HDL directly upregulates monocyte PDGFRβ, chemotaxis and TNFα production. Clin. Immunol. 137, 147–156. 10.1016/j.clim.2010.06.014 20637704 PMC2941543

[B34] Teslovich T. M. Musunuru K. Smith A. V. Edmondson A. C. Stylianou I. M. Koseki M.. (2010). Biological, clinical and population relevance of 95 loci for blood lipids. Nature 466, 707–713. 10.1038/nature09270 20686565 PMC3039276

[B35] Toth P. P. Barter P. J. Rosenson R. S. Boden W. E. Chapman M. J. Cuchel M.. (2013). High-density lipoproteins: a consensus statement from the National Lipid Association. J. Clin. Lipidol. 7, 484–525. 10.1016/j.jacl.2013.08.001 24079290

[B36] Vasseur E. Boniotto M. Patin E. Laval G. Quach H. Manry J.. (2012). The evolutionary landscape of cytosolic microbial sensors in humans. Am. J. Hum. Genet. 91, 27–37. 10.1016/j.ajhg.2012.05.008 22748209 PMC3397270

[B38] Xiong S. L. Liu X. Yi G. H. (2014). High-density lipoprotein induces cyclooxygenase-2 expression and prostaglandin I-2 release in endothelial cells through sphingosine kinase-2. Mol. Cell. Biochem. 389, 197–207. 10.1007/s11010-013-1941-y 24385109 PMC3950625

[B39] Zurawek M. Fichna M. Januszkiewicz-Lewandowska D. Gryczyñska M. Fichna P. Nowak J. (2010). A coding variant in NLRP1 is associated with autoimmune Addison's disease. Hum. Immunol. 71, 530–534. 10.1016/j.humimm.2010.02.004 20152874

