# Supplementary material for: Genetic analysis of long-lived families reveals novel variants influencing high density-lipoprotein cholesterol
Source: Front Genet. 2014 Jun 3;5:159. doi: 10.3389/fgene.2014.00159 (PMC4042684; doi:10.3389/fgene.2014.00159)
Supplement: Supplementary file 1 [file Presentation1.PDF]

Supplementary Material

Genetic Analysis of long-lived families reveals novel variants influencing high density-lipoprotein cholesterol

Mary F. Feitosa[1], Mary K. Wojczynski[1], Robert Straka[2], Candace M. Kammerer[3], Joseph H. Lee [4], Aldi T. Kraja[1], Kaare Christensen[5,6], Anne B. Newman[7], Michael A. Province[1], Ingrid B. Borecki[1],*

[1] *Division of Statistical Genomics, Department of Genetics, Washington University School of Medicine, St. Louis, MO, USA*
[2] *Department of Experimental and Clinical Pharmacology, College of Pharmacy, University of Minnesota, MN, USA*
[3] *Departments of Epidemiology and of Human Genetics, and Center for Aging and Population Health University of Pittsburgh, Pittsburgh, PA, USA*
[4] *Sergievsky Center and Taub Institute, College of Physicians and Surgeons, Columbia University, New York, NY, USA*
[5] *The Danish Aging Research Center, Epidemiology, University of Southern Denmark, Odense, Denmark*
[6] *Department of Clinical Genetics and Department of Clinical Biochemistry and Pharmacology, Odense University Hospital, Odense, Denmark,*
[7] *Department of Epidemiology, University of Pittsburgh Graduate School of Public Health, Pittsburgh, PA*

**Correspondence: Mary F. Feitosa, Division of Statistical Genomics,* Department of Genetics, Washington University School of Medicine; 4444 Forest Park Blvd; St. Louis, MO, 63108-2212, Campus Box 8506, USA
e-mail: mfeitosa@wustl.edu

SUPPLEMENTAL FIGURE 1 | The distribution of mean levels of HDL

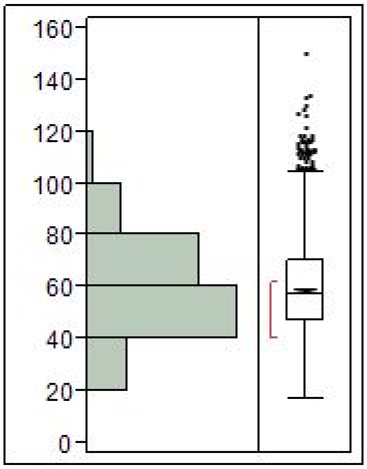

Moments

Mean	59.14001
Std Dev	17.305899
Std Err Mean	0.2698125
Upper 95% Mean	59.668988
Lower 95% Mean	58.611031
N	4114

SUPPLEMENTAL FIGURE 2 | Quantile-quantile plot of genome-wide association (observed $-\log_{10}$ (p−value) *versus* x expected $-\log_{10}$ (p−value) in abscissa) for HDL (λ_{GC}=1.03, SE=3.06 x10^{-5}). The results (-log10 p) include SNPs with acceptable imputation quality (r^2 MACH > 0.3) and with effect allele frequency between 1-99%.

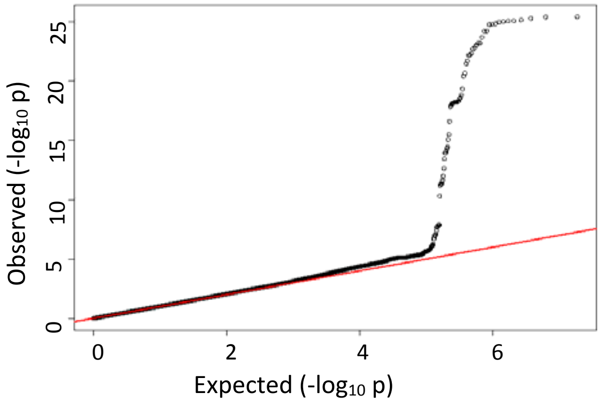

SUPPLEMENTAL TABLE 1 | Genome-wide associations (p-value < 1.0E-08) for plasma levels of HDL

SNP	Chr	Location	Gene	Function	CA	ACA	CAF	IP	N	β	SE	P
rs964184	11	116648917	near-ZNF259		C	G	0.86	1	4114	0.17	0.03	9.66E-08
rs11604424	11	116651115	ZNF259	intron	T	C	0.78	0	4113	0.15	0.03	4.00E-08
rs3741298	11	116657561	ZNF259	intron	T	C	0.79	1	4114	0.16	0.03	2.04E-08
rs12450571	17	5636654	near-NLRP1		G	C	0.47	0	4110	0.13	0.02	1.82E-08
c17b5639195INDEL	17	5639195	near-NLRP1			Insertion	0.47	1	4114	0.13	0.02	1.30E-08
kgp2603641	17	5639197	near-NLRP1		C	T	0.47	0	4114	0.13	0.02	1.39E-08
rs8080616	17	5643318	near-NLRP1		T	C	0.46	1	4114	0.13	0.02	9.78E-08
rs2215496	17	5646564	near-NLRP1		C	T	0.49	0	4113	0.12	0.02	7.16E-08
rs9989419	16	56985139	near-HERPUD1-CETP		G	A	0.60	0	4108	0.19	0.02	8.29E-16
rs193695	16	56985156	near-HERPUD1-CETP		G	A	0.65	0	4113	0.19	0.02	1.10E-14
rs72786786	16	56985514	near-HERPUD1-CETP		A	G	0.32	1	4114	0.28	0.03	3.64E-26
rs12448528	16	56985555	near-HERPUD1-CETP		G	A	0.78	0	4111	0.19	0.03	2.11E-12
rs7203286	16	56986762	near-HERPUD1-CETP		G	T	0.56	1	4114	0.22	0.02	2.11E-21
rs12446515	16	56987015	near-HERPUD1-CETP		T	C	0.32	1	4114	0.26	0.02	1.59E-25
rs56156922	16	56987369	near-HERPUD1-CETP		C	T	0.32	1	4114	0.26	0.02	1.65E-25
rs56228609	16	56987765	near-HERPUD1-CETP		T	C	0.31	1	4114	0.26	0.02	6.70E-26
rs173539	16	56988044	near-HERPUD1-CETP		T	C	0.33	1	4114	0.25	0.02	5.80E-25
rs247616	16	56989590	near-HERPUD1-CETP		T	C	0.32	1	4114	0.26	0.02	8.02E-26
rs12923459	16	56989830	near-HERPUD1-CETP		G	A	0.56	1	4114	0.22	0.02	3.71E-21
rs247617	16	56990716	near-HERPUD1-CETP		A	C	0.32	1	4114	0.26	0.02	9.26E-26
rs183130	16	56991363	near-HERPUD1-CETP		T	C	0.32	0	4113	0.25	0.02	1.43E-25
rs28888131	16	56991624	near-HERPUD1-CETP		G	A	0.81	0	4114	0.20	0.03	4.66E-12
rs12934632	16	56991741	near-HERPUD1-CETP		C	T	0.81	1	4114	0.20	0.03	4.66E-12
rs12920974	16	56993025	near-HERPUD1-CETP		G	T	0.72	0	4059	0.17	0.03	4.78E-11
rs12149545	16	56993161	near-HERPUD1-CETP		A	G	0.31	1	4114	0.26	0.02	3.75E-26
rs12708967	16	56993211	near-HERPUD1-CETP		T	C	0.81	1	4114	0.20	0.03	4.66E-12
rs3764261	16	56993324	near-HERPUD1-CETP		T	G	0.32	0	4111	0.26	0.02	7.63E-26
rs821840	16	56993886	CETP	upstream	G	A	0.28	1	4114	0.28	0.03	5.94E-25
rs36229786	16	56993901	CETP	upstream	A	C	0.81	1	4114	0.23	0.03	3.47E-14
rs711751	16	56993909	CETP	upstream	C	A	0.55	1	4114	0.14	0.02	1.73E-08

rs12720918	16	56994212	CETP	upstream	T	C	0.72	1	4114	0.18	0.03	2.76E-12
c16b56994244INDEL	16	56994244				Deletion	0.32	1	4114	0.26	0.02	4.95E-26
rs17231506	16	56994528	CETP	upstream	T	C	0.32	1	4114	0.26	0.02	9.81E-26
rs1800775	16	56995236	CETP	upstream	A	C	0.49	0	4109	0.21	0.02	4.44E-20
rs3816117	16	56996158	CETP	intron	C	T	0.49	1	4114	0.21	0.02	1.45E-19
rs711752	16	56996211	CETP	intron	A	G	0.42	1	4114	0.21	0.02	2.92E-19
rs708272	16	56996288	CETP	intron	A	G	0.42	0	4113	0.21	0.02	5.02E-19
c16b56996645INDEL	16	56996645				Insertion	0.42	1	4114	0.21	0.02	2.40E-19
rs1864163	16	56997233	CETP	intron	G	A	0.75	1	4114	0.27	0.03	1.97E-24
c16b56997349INDEL	16	56997349				Deletion	0.75	1	4114	0.27	0.03	6.26E-24
rs9929488	16	56998572	CETP	intron	G	C	0.63	0	4114	0.20	0.03	7.76E-15
rs12720926	16	56998918	CETP	intron	G	A	0.42	1	4114	0.21	0.02	5.57E-19
rs7203984	16	56999258	CETP	intron	A	C	0.81	0	4114	0.29	0.03	6.57E-23
rs11508026	16	56999328	CETP	intron	T	C	0.42	0	4112	0.21	0.02	7.62E-19
c16b56999778INDEL	16	56999778				Insertion	0.82	1	4114	0.30	0.03	9.16E-24
rs8045855	16	57000696	CETP	intron	T	A	0.82	1	4114	0.30	0.03	3.27E-22
rs12720922	16	57000885	CETP	intron	G	A	0.81	0	4114	0.30	0.03	1.48E-23
rs118146573	16	57000938	CETP	intron	G	A	0.88	1	4114	0.29	0.04	3.05E-16
rs4784741	16	57001216	CETP	intron	T	C	0.42	1	4114	0.21	0.02	7.04E-19
c16b57001254INDEL	16	57001254				Deletion	0.82	1	4114	0.30	0.03	5.98E-24
c16b57001274INDEL	16	57001274				Insertion	0.41	1	4114	0.21	0.02	8.16E-19
rs12444012	16	57001438	CETP	intron	A	G	0.42	1	4114	0.21	0.02	6.51E-19
c16b57001579INDEL	16	57001579				Insertion	0.28	1	4114	0.23	0.03	3.50E-15
c16b57001580INDEL	16	57001580				Insertion	0.39	1	4114	0.19	0.02	4.68E-15
c16b57001581INDEL	16	57001581				Insertion	0.42	1	4114	0.21	0.02	6.16E-19
rs9926440	16	57002663	CETP	intron	G	C	0.71	1	4114	0.19	0.03	2.19E-13
rs9939224	16	57002732	CETP	intron	G	T	0.80	1	4114	0.29	0.03	1.85E-23
rs7205804	16	57004889	CETP	intron	A	G	0.42	0	4038	0.21	0.02	1.14E-18
rs1532625	16	57005301	CETP	intron	T	C	0.43	0	4096	0.21	0.02	5.61E-19
rs1532624	16	57005479	CETP	intron	A	C	0.43	1	4114	0.21	0.02	6.16E-19
rs11076175	16	57006378	CETP	intron	A	G	0.83	1	4114	0.30	0.03	4.46E-23
rs7499892	16	57006590	CETP	intron	C	A	0.82	0	4112	0.30	0.03	5.88E-23
rs289713	16	57006829	CETP	intron	A	T	0.82	1	4114	0.29	0.03	1.92E-22

rs11076176	16	57007446	CETP	intron	T	G	0.83	0	4105	0.27	0.03	1.47E-18
rs289714	16	57007451	CETP	intron	T	C	0.82	0	4106	0.26	0.03	2.46E-17
c16b57009657INDEL	16	57009657				Insertion	0.59	1	4114	0.16	0.03	1.29E-08
rs117427818	16	57010486	CETP	intron	C	T	0.96	1	4114	0.47	0.06	1.14E-14
rs5880	16	57015091	CETP	missense	G	C	0.95	1	4114	0.39	0.06	6.50E-12
rs1800777	16	57017319	CETP	missense	G	A	0.96	0	4114	0.45	0.06	9.97E-13

Chr: chromosome; Location: in Megabase using build 37.3; CA: coded allele; ACA: alternative coded allele; CAF: coded allele frequency; IP:Imputed SNP is represented as “Y” (“N” = typed); β: regression coefficient for the coded allele; SE: Standard error of the β; P: P value of association

SUPPLEMENTAL TABLE 2 | Summary of SNP functional annotations from the ENCODE

SNPs	Chr	Position (hg19)	DNAse	Histone marks	Proteins bound	Regulatory chromatin states (ENCODE or Roadmap)	Motifs changed	Genes
rs12450571	17	5636654	n.a.	H3k09me3, H4k20me1, H3k27me3, H2az, H3k9me1	IKZF1	HUES6 Cell Line, ES-I3 Cell Line, iPS-15b Cell Line	SEF-1	Near- *NLRP1*
rs8080616	17	5636654	n.a.	H3k09me3, H4k20me1, H3k27me3, H2az, H3k9me1	n.a.	n.a.	Foxj1, Foxa, Foxk1, Foxo, Foxp1, Foxl1,HDZC2, HMG, Irf, Nanog, Sox, p300	Near- *NLRP1*
rs2215496	17	5646564	n.a.	H3k09me3, H4k20me1, H3k27me3, H2az, H3k9me1	n.a.	n.a.	Foxp1, Pou2f2	Near- *NLRP1*
rs3741298	11	116657561	Medulloblastoma	H4k20me1, H3k9ac, H2az, H3k9me1, H3k36me3, H3k27ac, H3k79me2, H3k04me1,	BCL11A, EBF1, EPAX5N19, PU1, SP1, TCF12, CTCF	B-lymphocyte, lymphoblastoid, hepatocellular carcinoma, leukemia, skeletal muscle myoblasts, lung fibroblasts, epidermal keratinocytes, mammary epithelial cells, umbilical vein endothelial cells, etc.	CDP, GATA, GR, Sox, Hoxb3, NR4A, RFX5, Pou3f2, Cutl1	ZNF259
rs72786786	16	56985514	B-lymphocyte lymphoblastoid, B cell, promyelocytic leukemia cells, neonatal dermal fibroblasts, primary Th2 T cells	n.a.	SPI1, SP1, ZNF263, BCL11A, EBF1, PAX5, TCF12, CTCF	B-lymphocyte, lymphoblastoid, hepatocellular carcinoma, etc.	GATA, Myf, SRF, UF1H3BETA	HERPUD1-CETP

n.a.: not available in ENCODE
